# Metagenomic Analysis of DNA Viruses with Targeted Sequence Capture of Canine Lobular Orbital Adenomas and Normal Conjunctiva

**DOI:** 10.3390/microorganisms11051163

**Published:** 2023-04-28

**Authors:** Elizabeth A. F. Schaefer, Shirley Chu, Kristine M. Wylie, Todd N. Wylie, Obi L. Griffith, Jacqueline W. Pearce, Gayle C. Johnson, Jeffrey N. Bryan, Brian K. Flesner

**Affiliations:** 1 Department of Veterinary Medicine and Surgery, College of Veterinary Medicine, University of Missouri, Columbia, MO 65211, USA; 2 McDonnell Genome Institute, Washington University, St. Louis, MO 63108, USA; 3 Department of Pediatrics, Washington University, St. Louis, MO 63110, USA; 4 Department of Medicine, Washington University, St. Louis, MO 63110, USA; 5 Department of Veterinary Pathobiology, University of Missouri, Columbia, MO 65211, USA

**Keywords:** canine lobular orbital adenoma, papillomavirus, parvovirus, virome, canine, conjunctiva, ViroCap, ViroMatch

## Abstract

Our study aims are: (1) to evaluate phenotypically normal canine conjunctival and orbital tissue and tissue from canine lobular orbital adenomas (CLOAs) for the presence of viral genomic material and (2) phylogenetically classify detected DNA viruses to determine if a DNA virus is associated with CLOAs. A total of 31 formalin fixed paraffin embedded CLOA tissue samples, 4 papillomas or sarcoid, and 10 fresh clinically normal conjunctival tissues were included in this study. Genomic DNA was isolated from all samples and sequencing libraries were prepared. The libraries were molecularly indexed and pooled and viral DNA was enriched via targeted sequence capture utilizing ViroCap. The libraries were sequenced on the Illumina HiSeq platform and compared to known viral DNA reference genomes to identify viral DNA. Carnivore parvovirus was identified in 6.4% and 20% of CLOA tissue and normal conjunctival samples, respectively. This study showed that conjunctival tissue from healthy dogs and CLOAs uncommonly harbor DNA viruses, and no DNA virus was associated with these tumors. Further studies are needed to evaluate the etiologic cause of CLOAs.

## 1. Introduction

Canine lobular orbital adenomas (CLOAs) occur in middle age to older dogs and can affect a variety of breeds. They are of epithelial origin and thought to derive from either the lacrimal gland or less likely the zygomatic salivary gland or gland of the third eyelid [1]. They may affect the third eyelid, conjunctiva, or eyelids and extend into the orbit surrounding the globe [1,2]. These tumors are benign, typically lobulated and friable, and may be nodular or solid. The tissue consists of well-differentiated glandular tissue that is lobulated and lacks ducts with a mucoserous secretory pattern [1]. They tend to be locally aggressive and have been shown to occur bilaterally in 13% of cases [1].

While affected dogs typically have a normal globe, these tumors can result in ophthalmic complications such as exposure keratitis and reduced vision secondary to third eyelid elevation or mass occupation in severe cases. Surgical removal of these tumors is challenging and recurrence rates are high [1]. Therefore, procedures such as enucleation or exenteration are sometimes necessary to ensure complete excision. These procedures are not ideal, especially in an otherwise visual eye as they result in blindness [1]. Further investigation into the underlying etiology of these tumors is warranted, specifically microbial, viral, and genetic triggers. A better comprehension of the etiology and pathogenesis of these tumors could promote development of preventative measures and less invasive therapies.

The skin and mucosal surfaces of mammalian species are colonized by a multitude of bacteria and viruses [3,4,5,6,7,8,9]. Alterations in the normal bacterial or viral microbiome can lead to disease, including infection, inflammation, or cancer transformation [3,8,10,11,12,13,14]. Specifically, several DNA viruses have been associated with human and veterinary tumors, including human and animal papillomaviruses [15,16,17,18,19,20,21,22,23], animal herpes viruses [24,25], human herpesvirus 8 [26], Epstein–Barr virus [27,28], hepatitis B virus [29], hepatitis delta viruses [30,31,32], and Merkel cell polyomavirus [14,33,34]. Therefore, investigation into viral etiologies of neoplastic tissue is warranted. To the authors’ knowledge, an underlying cause for CLOAs has not been determined. In the human literature, there are a variety of benign lacrimal and salivary gland tumors. Although a viral etiology has not been conclusively elucidated in these tumors, high risk human papillomaviruses (HPV) have been detected in 35% of human salivary gland tumors [35]. Therefore, it is sensible to evaluate canine orbital tumors for the presence of viral DNA.

The ocular viral microbiome or virome has been minimally studied in human patients [36] and not at all in veterinary patients. Currently, the virome in canine conjunctival tissue is unknown. While various studies have evaluated specific viruses present on the ocular surface using traditional viral identification techniques, such as culture, serology, or targeted nucleic acid-based testing, including polymerase chain reaction (PCR), these techniques are limited to evaluation of a single or few specific viruses [37,38,39,40,41]. Currently, there are over 140 families, 80 genera, and 4000 species of known viruses (International Committee on Taxonomy of Viruses) [42]. Therefore, detection of these viruses requires more advanced technologies. Next generation sequencing significantly increases the sensitivity of viral detection as it allows for accurate and efficient sequencing of an entire genome [43].

A sensitive and specific method to detect viral genomic material in normal and neoplastic tissue is desirable but historically difficult to achieve. There is difficulty with virus detection in clinical samples because viruses have exceedingly variable and complex genomes, they lack a conserved anchor sequence as is present in bacteria, and often the viral load is low in clinical samples [43]. A metagenomic shotgun sequencing (MSS) approach has the potential to be more successful in agnostically identifying viral sequences in tissue samples when compared to traditional viral detection techniques. ViroCap is a targeted sequence capture method, in which MSS samples are enriched for viral nucleic acids using oligonucleotide biotinylated probes targeting a comprehensive panel of known viral reference genomes [44]. The probe set includes over 2 million probes which capture viral sequences from over 34 viral families, 190 genera, and 337 species of viruses known to infect vertebrates [44]. This technology has been used to detect viruses from tumor tissues from cats with oral squamous cell carcinoma [45], vaginal swabs from women with preterm births [46], nasopharyngeal secretions, plasma, stool, whole blood, cerebrospinal fluid, tracheal aspirates, and skin swabs from children [44,47], cloacal swabs from a mass die off of Canada geese [48], and tissues from lab and pet mice [49].

Our group previously investigated canine papillomavirus as a cause in CLOAs, due to the association of papillomaviruses in human glandular tissues [35,50,51]. Papillomavirus DNA was not detected via PCR in CLOA samples [52]. Therefore, we aimed to expand the breadth of our evaluation to determine if any DNA virus or retrovirus was present in tissue from CLOAs. Further, we sought to evaluate clinically normal canine conjunctival and orbital tissue for the presence of DNA viruses by use of a comprehensive viral sequencing method. We hypothesized that viral DNA would be overrepresented in CLOA samples.

## 2. Materials and Methods

### 2.1. Phenotypically Normal Control Group

Ten normal control dogs, approximately 2–12 years of age, with clinically normal orbital tissues were identified at the local animal shelter and the University of Missouri Veterinary Health Center and euthanized for reasons unrelated to this study. Seven of these dogs were clinically healthy. The remaining three dogs had a history of hypoadrenocorticism or cancer. Table S1 describes the clinical characteristics and viral read counts for each sample. An anterior segment and adnexal examination was performed prior to euthanasia in each dog by one author (E.S.). An excisional biopsy was aseptically collected from the superior and inferior conjunctival fornix of each eye. Freshly sterilized instruments were used for each eye. Following tissue collection, fresh tissue was flash frozen in liquid nitrogen and stored at −80 °C until DNA extraction. All tissue collections for normal conjunctival samples occurred post-mortem after obtaining informed consent from the owner and all papillomavirus control and CLOA tissues used in the study were collected as part of the patient’s routine care. Tissue collection was within the guidelines of exempt protocol #9866 which was approved by the University of Missouri Animal Care and Use Committee.

### 2.2. Canine Lobular Orbital Adenoma Group

Formalin fixed paraffin embedded (FFPE) tissue samples from 33 dogs (36 eyes) were acquired from COPLOW (Table S1). Two samples did not yield sufficient DNA of sufficient quality and quantity for sequencing. The histopathologic description provided by Headrick et al. was used to confirm all samples as CLOAs [1].

### 2.3. Papillomavirus Positive Control Group

Three FFPE tissue samples, two from canine papillomas and one from an equine sarcoid, and one fresh frozen tissue sample from a canine papilloma, were obtained for use as positive controls, Table S1. The presence of papillomavirus in the papillomavirus positive control group was also evaluated with PCR and IHC. Results are presented in Table S2 and PCR conditions are outlined in Table S3. The IHC methods are included below.

### 2.4. DNA Extraction

Each FFPE tissue block was sectioned into 10 µm tissue scrolls. The DNeasy Blood and Tissue DNA Extraction Kit (Qiagen, N.V., Hilden, Germany; 69504) was used for the fresh tissue samples and the QIAmp DNA FFPE Tissue Kit (Qiagen; 56404) was used for the FFPE samples. The Qubit 2.0 fluorometer (Invitrogen, Life Technologies, Carlsbad, CA, USA) was used to quantify extracted genomic DNA following the manufacturer’s protocol for the Quant-iT dsDNA HS Assay. Additionally, the NanoDrop 2000 Spectrophotometer (Thermo Fisher Scientific, Waltham, MA, USA) was used to assess DNA quantity and purity following the manufacturer’s protocol. The DNA extractions were repeated until 3 µg of total pooled DNA was obtained, and DNA was stored at −20 °C following extraction.

### 2.5. Library Preparation, DNA Capture, and Nucleic Acid Sequencing

Following DNA extraction, genomic DNA was submitted to the McDonnell Genome Institute at Washington University (St. Louis, MO, USA). The Swift Biosciences Accel NGS kit with dual same indexes was used to construct DNA sequencing libraries. Prior to sequencing, the targeted sequence capture panel, ViroCap, synthesized by Roche NimbleGen, was used to enrich viral nucleic acid [44]. The Roche NimbleGen procedures were followed for targeted sequence capture. Briefly, libraries from the fresh frozen samples were pooled and hybridized with the custom probe set for 72 h at 47 °C. The libraries from the FFPE samples were pooled and hybridized together but separately from the fresh frozen samples. The samples were then washed and the two library pools were normalized and pooled together.

Libraries were sequenced on the Illumina HiSeq 4000 instrument (Illumina, San Diego, CA, USA) as 2 × 150 bp reads to an average of 1.9 Gb per library.

### 2.6. Sequence Analysis with ViroMatch

Sequence analysis was done as previously described [45,53] with the exception that host screening utilized the canis_lupus_familiaris_3.1/canfam3 or equus_caballus_3.0/equCab3 reference genomes. Briefly, reads filtered against the host reference genome prior to viral assessment. Resulting reads are screened for putative viral reads by nucleotide mapping (BWA-MEM) and translated mapping (Diamond) against a database of virus-specific reference genome sequences collected from NCBI GenBank. The putative viral hits are subsequently mapped to the NCBI nucleotide (nt) and NCBI nonredundant (nr) amino acid databases. Ambiguous hits, including those that map similarly to viruses, human, bacteria, and to repetitive regions are excluded. Average genomic coverage (depth of coverage or DoC) was estimated from base representation by the extracted, deduplicated, and aligned reads for each base of the genome. Breadth of coverage (BoC) was estimated in SAMtools and was defined as the percentage of bases of the reference viral genome that was covered by sequence reads at a level of 5× or higher. Sequence data for the samples were deposited into the Sequence Read Archive (SRA) under BioProject PRJNA718631.

### 2.7. Papillomavirus Immunohistochemistry

The positive control samples were evaluated for papillomavirus via IHC prior to nucleic acid sequencing. Biopsy specimens were fixed by immersion in neutral buffered 10% formalin. Specimens were sampled for examination and dehydrated, then embedded in paraffin by standard methods. Sections of 5 µm were prepared and stained by hematoxylin and eosin (HE). Additional 5 µm thick specimens were prepared and placed on glass slides, then deparaffinized and rehydrated by immersion in xylene and graded alcohols to distilled water. Staining (Biocare Medical; Pacheco, CA, USA; IPS0001) was done in a Biocare IntelliPATH FLX autostainer. Sections were then treated with 3% hydrogen peroxide for 15 min to block endogenous peroxidase activity and washed in buffer. Non-specific reagent binding was blocked by treating with Sniper (Biocare Medical; Pacheco, CA, USA; BS966) for 10 min with a second buffer wash. Slides were treated with mouse monoclonal anti-papillomavirus antibody which is a mixture of the BPV-1/1H8 against SDS disrupted BPV-1 and CAMVIR-1 against L1 of HPV-16 (Abcam; Waltham, MA, USA; ab2417) at a dilution of 1:600 for 30 min, rinsed with buffer, and treated with anti-mouse HRP EnVision+ (Agilent DAKO; Santa Clara, CA, USA; K4001) for 30 min. After rinsing with distilled water Romulin Red chromogen (Biocare Medical; Pacheco, CA, USA; RAEC810) was applied and CAT hematoxylin (Biocare Medical; Pacheco, CA, USA; CATHE) applied as a counter stain. After a final rinse, tissues were dehydrated with graded alcohols and xylene and coverslipped. For each specimen, a second slide was stained with an irrelevant antibody to demonstrate the specificity of staining and a positive control was positively and negatively stained for each run.

### 2.8. Reporting Thresholds

Based on previous studies evaluating index swapping with the Illumina platform and targeted sequence capture [47,54,55,56], a threshold for each virus was established to eliminate false positives. The threshold to confirm that a specific virus was present in the sample was set at 0.1% of the total reads for that virus in the appropriate pool [47]. To establish the number of sequence reads reported, the calculated threshold number of reads was subtracted from the raw observed number of reads for that virus within a sample. After subtraction of the threshold, a virus was considered to be present and detected within a sample if the reported number of sequence reads was ≥1 [47,54,55,56]. Additionally, a virus was considered to be present and detected if there was ≥0.5× DoC [45]. Exceptions to this rule included samples that were manually reviewed for the presence of reads that were distributed across the viral genome to confirm presence of a virus. Statistical comparison for the presence of a virus between the CLOA and normal control groups was done with the two-tailed Fischer exact test.

### 2.9. Parvovirus Polymerase Chain Reaction

The presence of parvovirus and subtyping were confirmed with PCR. A set of primers that amplify the entire VP2 region were used [57]. Non-template samples served as negative controls and a canine parvovirus patient isolate that was used as a positive control for commercial canine parvovirus detection at the University of Missouri Veterinary Diagnostic lab was used a positive control. The conditions for the reaction can be found in Table S4. PCR amplicons were purified (Qiagen; 28104) according to the manufacturer’s protocol. Amplicons were submitted to the University of Missouri Genomics Technology Core for forward and/or reverse Sanger sequencing.

### 2.10. Merkel Cell Polyomavirus Polymerase Chain Reaction

The presence of Merkel Cell Polyomavirus was confirmed with PCR. A set of primers, LT3, that amplify the large and small T antigen region was used [58]. Non-template samples served as negative controls and plasmids containing the large and small T antigen region from isolate MCC339 and a wild type isolate were used as positive controls. The plasmids were obtained through the NIH HIV Reagent Program, Division of AIDS, NIAID, NIH: pcDNA6.Tag206.V5(2B4), ARP-11931, and pcDNA3.MCV339 (144-3696), ARP-11930; contributed by Dr. Patrick Moore. The conditions for the reaction can be found in Table S4. PCR amplicons were purified and Sanger sequenced as above.

## 3. Results

### 3.1. Samples

Ten dogs euthanized for non-ocular pathologies were used in the phenotypically normal control group. For the CLOA group, the affected eye, breed, age, and gender of each of the 31 dogs is provided in Table S1. The average age was 10.5 years (range 6–16 years). The right eye was affected in 11 cases, the left eye in 16 cases, and both eyes in 4 cases; one case was presumed to be bilateral based on follow-up, however, the second eye was not submitted for histopathologic evaluation.

### 3.2. Summary of DNA Viruses Detected in CLOAs and Normal Canine Conjunctiva

**Figure 1:**
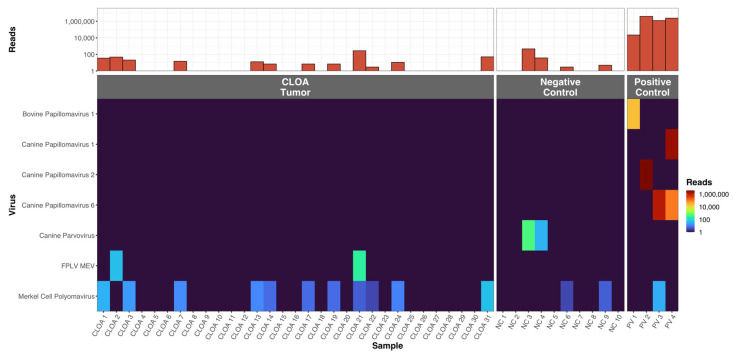
Summary of the viruses detected by ViroCap from each patient in the CLOA and positive and negative control groups. The top bar graph shows the number of viral reads in each sample. The heatmap shows the number of reads of each virus in each sample. FPLV = feline panleukopenia virus. MEV = mink enteritis virus.

Carnivore parvoviruses were detected in 6.4% (2/31) and 20% (2/10) of CLOA and normal conjunctiva, respectively (*p* = 0.25), Figure 1. Papillomaviruses were detected in all of the positive control samples. Merkel Cell Polyomavirus or a related virus was detected in 38.7% (12/31) and 20% (2/10) of CLOA and normal conjunctiva tissue samples, respectively (*p* = 0.45).

### 3.3. Parvovirus

**Figure 2:**
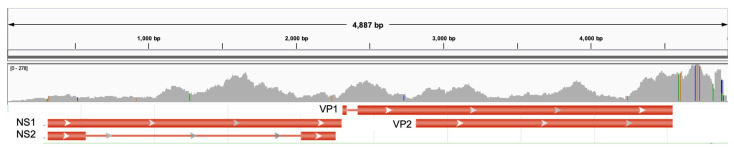
NC3 had the most reads that aligned to parvovirus. The 4921 bp canine parvovirus 2a (KY403998.1) genome is annotated (GenBank) and shown above the reads that aligned to this genome. This new strain was deposited into GenBank as MZ647470. The VP2 antigen is used to differentiate viral subtypes. NS = non-structural. Colored vertical lines on the gray coverage track represent single nucleotide variants compared with the reference.

Parvovirus (canine parvovirus, FPLV/MEV) was detected in 8.9% (4/45) of all of the samples; 6.4% (2/31), 20% (2/10), and 0% (0/4) of CLOA, normal conjunctiva, and papillomavirus positive control group, respectively. The available medical records for these dogs did not describe a history of suspected parvoviral infection. Table S5 shows the closest viral match and coverage. VP2, viral capsid, is the major antigenic protein and determines viral tissue tropism and host range. VP2 is thus used to identify variants. Small differences in amino acid sequence differentiate the variants (e.g, residue 426 is Asn in CParvoV-2a, Asp in CParvoV-2b, and Glu in CParvoV-2c). The results from the parvovirus VP2 PCR and resulting amino acid translations are presented in Table 1. Based on these key amino acid differences, CParvoV-2a and CParvoV-2b were identified in the normal conjunctival samples. In the current study, a new strain of the new CParvoV-2a variant was identified in patient NC3, Figure 2. This new strain was deposited into GenBank as MZ647470. The same strain was identified in both the left and right eye in NC3. Similarly, the SNVs that were identified in the CParvoV-2b genome in the left eye from patient NC4 were identified in the right eye. Either feline panleukopenia virus (FPLV) or mink enteritis virus (MEK) were detected in CLOA2 and CLOA21. The gaps in coverage and marked similarity in sequence between FPLV and MEK prevented identification of this virus as either FPLV or MEK.

### 3.4. Papillomavirus

The positive control samples PV1, PV3, and PV4 were sequenced in a previous ViroCap study [45] and as expected, the consensus papillomavirus sequences were identical to the previous study. Papillomavirus was also detected in PV1-PV4 via PCR or IHC. PV1, PV3, and PV4 were classified as CPapV-1 (GenBank Accession KY825186), CpapV-6 (GenBank Accession KY802017), and BPV-1 (GenBank Accession KY886226), respectively. An additional control sample from a canine papillomatous lesion was added to this study, PV2. Tables S6 and S7 show the papillomavirus strain that was detected and the coverage for the positive control samples. PV2 was confirmed to have papillomavirus via IHC for L1, PCR, and ViroCap, Table S2. The complete genomic sequence of the CPapV-2 from PV2 was submitted to GenBank as MW881228, Figure S1 and Tables S8 and S9.

### 3.5. Merkel Cell Polyomavirus (MCPyV)

Merkel Cell Polyomavirus or a related virus was the most commonly detected virus and was identified in 20% (2/10) and 35% (11/31) of normal conjunctiva and CLOA tissue samples, respectively (*p* = 0.46). Table S10 shows the closest viral match and coverage. The DoC and BoC were low, 0.1–1.4× and 0–3.69% respectively, Figure S2. PCR and Sanger sequencing were done in the samples that contained the most MCPyV reads, CLOA31, CLOA1, PV3, and CLOA3, Figure S3. These samples included the genomic DNA (gDNA) samples that were submitted for library generation and also included the CLOA31 library and genomic DNA from CLOA3 that was previously isolated as a backup and not submitted for library generation. The libraries for CLOA1, PV3, and CLOA3 were exhausted during sequencing and thus could not be tested. PCR and Sanger sequencing confirmed the presence of MCPyV or a related virus in the CLOA31 library, submitted CLOA3 gDNA and PV3 gDNA samples. MCPyV was not found in the backup CLOA3 gDNA sample that was not submitted for library generation and sequencing. An amplicon was not consistently seen in the PCR duplicate samples which is likely due to the low amount of MCPyV genetic material in these samples, Figure S3, consistent with previous PCR validation experiments [45]. The PCR amplicon sequence was identical in all of these samples and on BLAST analysis was 100% identical to the wild type Merkel Cell Polyomavirus and to pcDNA6.TAg206.V5(2B4). The only known canine polyomavirus is a betapolyomavirus, Canis familiaris polyomavirus 1 (GenBank NC_034456.1). When all of the sequenced reads from the sample with the highest MCPyV read count, CLOA31, were aligned to Canis familiaris polyomavirus 1, only 13 reads were identified with a DoC and BoC of 0.1× and 0.27%, respectively. The polyomavirus detected in the canine samples was thus more closely related to MCPyV than to Canis familiaris polyomavirus 1. The presence of MCPyV or a related virus could be explained by a true infection in these dogs or contamination introduced into the samples during patient or sample handling. Support for the latter include, (1) the BoC and DoC were low in most samples, (2) MCPyV has not been previously found in dogs, (3) the same sample from one of the subjects in this study, PV3, was also used in a previous study [45] and reads aligning to MCPyV were not found, (4) MCPyV are considered commensal in the skin of people [59,60], and (5) MCPyV reads were not found in the CLOA3 gDNA sample that was not submitted for library generation.

## 4. Discussion

To the authors’ knowledge, this is the first study to report the virome of canine conjunctival tissue. To date, there is only one study that evaluates the viral microbiome of canine tumor tissue [61] and viruses were not confidently identified in the common canine tumors that were evaluated. In this study, DNA viruses were infrequently identified in normal canine conjunctival tissue. Additionally, no single DNA virus was associated with CLOAs in the present study.

Canine parvovirus (CParvoV) was not associated with CLOA but it was detected in 8.7% of dogs who were asymptomatic for parvovirus infection. True infections were suspected since (1) the BoC and DoC were relatively high in some samples, (2) the VP2 sequence in the different eyes from the same dog and from different DNA extractions were the same, (3) different strains were seen in different dogs, and (4) temporally dispersed samples were affected. Parvoviruses are small single stranded non-enveloped DNA viruses. Canine parvovirus is a member of the Parvoviridae family, genus *Protoparvovirus*. The *Protoparvovirus* genus [62] includes the canine, feline, and mink parvoviruses which cause retinal dysplasia and congenital cerebellar hypoplasia in cats [63], gastrointestinal disease in dogs and cats, myocarditis in dogs [64], severe interstitial pneumonia in mink kits, and chronic Aleutian disease in adult mink [65]. The carnivore parvoviruses are transmitted via the fecal-oral route or from contaminated surfaces. After infection, the virus typically affects cells with high mitotic activity, including intestinal epithelium, lymphoid tissue, and bone marrow [64]. In addition to the intestinal epithelium, CParvoV has been identified in the epithelial tissues of the esophagus, tongue and skin, spleen, liver, kidney, heart, and brain [66,67,68]. Human parvovirus B19, a member of the genus *Erythrovirus*, has been identified in normal lacrimal and orbital connective tissues in humans and is associated with uveitis in humans [69,70,71,72]. To the authors’ knowledge, CPapV, FPLV, or MEV have not been detected in conjunctival tissues.

For the CLOA samples with MEV/FPLV, recent vaccination as the cause was not possible, since a licensed vaccine with MEV does not exist in the US. Feline panleukopenia virus would not be present in a licensed canine vaccine; moreover, a feline vaccine would not have been used to vaccinate a dog. CLOA2 and CLOA21 were collected 9 years apart and had different strains of the virus. Alternatively, FPLV/MEV may have been replicating transiently in the dogs without causing disease or virus shedding. In an experimental infection of dogs with FPLV, FPLV replicated in the thymus, spleen, and bone marrow but not in mesenteric lymph nodes or small intestine [73]. Low levels of FPLV were identified in the mesenteric lymph nodes in another experimental infection [74]. FPLV can also be found in symptomatic dogs that were also infected with CParvoV-2a [75]. The identification of FPLV/MEV in the canine samples in the current study could be due to transient infection, asymptomatic carrier status, or less likely contamination. FPLV/MEV or other members of the protovirus genus have not been shown to be tumorigenic in dogs or any other species and thus was not considered to be a cause of CLOA.

It is possible that the CParvoV-2b that was detected in NC4 was due to recent vaccination. Sequencing of the vaccines available in the US at the time of sample collection would be needed for comparison. Recent vaccination was unlikely to be the cause of CParvoV-2a detection in NC3, since sample NC3 was collected in 2017 and a vaccine that contains CParvoV-2a would not have been available in the US [76]. An asymptomatic carrier state was considered the most likely for NC3 since it was unlikely that the aseptic technique followed in this study resulted in environmental contamination in both the left and right conjunctiva. Carnivore parvoviruses are generally considered to be cleared within 10 days of infection and little residual viral DNA can be detected [77] but it is possible that virus could be detected in the conjunctiva more than 10 days post infection. NC3 and NC4 could have had an unrecorded history of parvoviral infection. Asymptomatic or chronic carriers of CParvoV-2 have not been previously described in dogs but have been seen in cats [78]. The clinical significance of identifying parvovirus is unknown since we do not know if these dogs were shedding virus and could thus transmit the disease. Further prospective studies are needed to evaluate the presence of parvovirus in ocular tissue and feces to confirm a canine carrier state.

Limitations of the present study include the moderately small sample size of both CLOA samples and phenotypically normal dogs. Further studies evaluating the virome of domestic species should consider including higher animal numbers, and positive controls with lower viral loads. Further, RNA viruses, unless reverse transcribed, would not have been identified with the methods used in this study. While an RNA virus is unlikely to be the underlying etiology of these tumors, it cannot be ruled out based on the present study. Another limitation is that the vast majority of tissues were from dogs that were euthanized and thus follow-up studies with these dogs were not possible.

## 5. Conclusions

In conclusion, the normal canine conjunctiva infrequently contained DNA viruses and a DNA virus was not associated with CLOA. Further research is needed to evaluate the etiology and pathogenesis of CLOAs.

## Figures and Tables

**Table 1:** Key SNVs in VP2 as determined by ViroCap and targeted PCR in the parvovirus genomes sequenced in this study that allowed subtyping.

Sample	5	80 ^a^	87	93 ^a^	101	103 ^a^	267	297	300	305	322	323 ^a^	324	334	341	370	375	390	426 ^b^	440 ^b^	555	564 ^a^	568 ^a^
NC3 ^c^	A	R	L	N	T	A	Y	A	G	Y	T	N	I	A	P	Q	D	T	N	A	V	S	G
CLOA21	A	K	M	K	T	V	F	S	A	D	T	D	Y	A	P	Q	D	T	N	N/A	V	N	A
NC4 ^d^	A	R	L	N	T	A	F	A	G	Y	T	N	Y	A	P	Q	D	T	D	T	V	S	G
CLOA2 ^e^	N/A	K	M	K	N/A	N/A	N/A	N/A	N/A	N/A	N/A	N/A	N/A	N/A	P	Q	N/A	N/A	N/A	N/A	N/A	N/A	N/A

N/A: no aligned reads at this location. ^a^ These residues generally discriminate between FPLV and CParvoV-2. ^b^ The criteria for subtyping CParvoV-2 at the University of Georgia veterinary diagnostic lab (personal communication) is as follows: CParvoV-2a = AAT (N); CParvoV-2b = GAT (D); CParvoV-2c=GAA (E) at residue 426; CParvoV-2c = ACA (T) at residue 440 but 440 has been shown to be variable. ^c^ The same sequence was identified with Sanger sequencing from a sample from the left and a sample from the right eye. ^d^ The same sequence was identified with Sanger sequencing from a sample from the left eye and 2 separate FFPE scroll preparations of tissue from the right eye. ^e^ This sample did not produce a PCR product with the canine parvovirus primers for VP2.

## Data Availability

Sequence data for the samples were deposited into the Sequence Read Archive (SRA) under BioProject PRJNA718631.

## Supplementary Materials

The following supporting information can be downloaded at: https://www.mdpi.com/article/10.3390/microorganisms11051163/s1, Table S1: Patient characteristics and read information for each sample that was sequenced. Table S2: Comparison of papillomavirus detection techniques with conventional PCR, IHC, and ViroCap for positive control samples. Table S3: FAP59/64 primer sequences and PCR conditions. Table S4: Parvovirus primer sequences for VP2 and PCR conditions. Table S5: Canine patients with parvovirus detected with ViroCap. Coverage metrics and closest Merkel cell polyomavirus strain information are presented. Table S6: Read counts for papillomavirus positive controls across all samples. One sample, PV4, had a read count that was above the 0.1% threshold and misalignment was suspected. Table S7: Coverage metrics and papillomavirus strains that were detected with ViroCap in the positive control samples. Table S8: Novel complete canine papillomavirus 2 genome discovered by ViroCap. Table S9: Variants and predicted functional outcome in the novel papillomavirus strain (GenBank MW881228) detected in PV2 compared to the reference canine papillomavirus 2 genome (NC_006564.1). Table S10: Canine patients with Merkel cell polyomavirus detected with ViroCap. Coverage metrics and closest Merkel cell polyomavirus strain information are presented. Figure S1: Aligned reads from the positive control sample PV2 that aligned to CPapV-2 (GenBank NC_006564.1) after deduplication are shown. This strain was deposited into GenBank as MW881228, Canine Papillomavirus 2, isolate Missouri, Isabelle, complete genome. Colored vertical lines on the gray coverage track represent single nucleotide variants compared with the reference. Figure S2: CLOA31 had the most reads that aligned to Merkel Cell Polyomavirus but the DoC and BoC were low. The 5343 bp Merkel Cell Polyomavirus (GenBank NC_010277.2) genome is annotated and shown above the reads that aligned to this genome. GP1 is the VP2 capsid protein. GP2 is the VP1 major capsid protein. GP3 is the large T antigen. The gene in the far right is the small T antigen. Figure S3: Merkel Cell Polyomavirus was detected by PCR and Sanger sequencing in the samples that contained the highest ViroCap Merkel Cell Polyomavirus read counts. The LT3 primer set was used. The PCR product was visualized on an 1% agarose gel as a 308 bp band. Lane 1: 100 bp ladder; lane 2: no template control; lanes 3–4: CLOA31 gDNA; lanes 5–6: CLOA31 library; lane 7: Merkel Cell Polyomavirus plasmid pcDNA6.TAg206.V5(2B4); lane 8: Merkel Cell Polyomavirus plasmid pcDNA3.MCV339 (144-3696); lanes 9–10: CLOA1 gDNA; lanes 11–12: CLOA3 gDNA that was submitted for library production and sequencing; lanes 13–14: CLOA3 gDNA that was not submitted for library production and sequencing; lanes 15–16: PV3 gDNA. These amplicons were subsequently sequenced via Sanger sequencing and confirmed the presence of Merkel Cell Polyomavirus in lanes 7–8 (Merkel Cell Polyomavirus positive control plasmids), lane 5 (CLOA31 library), lane 12 (CLOA3 gDNA submitted sample) and lanes 15–16 (PV3 gDNA). Reference [79] is cited in the Supplementary Materials.
