# Supplementary material for: Metagenomic Analysis of DNA Viruses with Targeted Sequence Capture of Canine Lobular Orbital Adenomas and Normal Conjunctiva
Source: Microorganisms. 2023 Apr 28;11(5):1163. doi: 10.3390/microorganisms11051163 (PMC10223289; doi:10.3390/microorganisms11051163)
Supplement: Supplementary file 1 [file microorganisms-11-01163-s001.zip › microorganisms-2327398-supplementary.pdf]

Supplementary Information

Index hopping resulting in misalignment

The samples in this study were sequenced on the HiSeq4000 with Exclusion Amplification (ExAmp) chemistry. Index hopping is known to occur at a higher rate on sequencing platforms that use ExAmp technology versus the traditional cyclical bridge-PCR of random clustering for cluster generation [1]. Index hopping occurs due to free barcoded adapter/index primers present in a multiplexed library pool. The free adapter can prime and extend reads in the same lane during the clustering step. The index hopped and extended strand is then free to seed another nanowell. This leads to mis-assignment of reads and the appearance of contamination. Index hopping mostly affects ExAmp workflows because ExAmp relies on cluster growth proceeding at a faster rate than cluster seeding instead of the wash step after read binding in bridge amplification. Index hopping can occur on average in 1% of reads [1]. In this study, the contaminating reads for each sample and virus were <0.1% of the total reads for each virus in the sequencing pool. Based on previous studies [2-5] these findings suggest that read misalignment occurred due to index swapping and occurred due to the exponentially higher levels of viral DNA in the positive control samples compared to the low input of the CLOA and negative control libraries and the low diversity of the input libraries.

Table S1: Patient characteristics and read information for each sample that was sequenced

#	Patient	Species	Breed	Age (years)	Sex	Lesion	Tissue Type[a]	Location[b]	Year Collected	Other Clinical Information	Viral reads[c]
1	CLOA1	Canine	Golden Retriever	12	MI	CLOA	FFPE	OS	2002	N/A	25,957
2	CLOA2	Canine	Shepherd mix	10	MN	CLOA	FFPE	OD	2003	N/A	14,409
3	CLOA3	Canine	German Shepherd mix	8	FS	CLOA	FFPE	OS	2003	N/A	3840

4	CLOA4	Canine	Samoyed	11	MN	CLOA	FFPE	OD	2005	N/A	25,057
5	CLOA5	Canine	Labrador Retriever	11	MN	CLOA	FFPE	OS	2005	N/A	23,334
6	CLOA6	Canine	Boston Terrier	8	FS	CLOA	FFPE	OS	2007	N/A	16,257
7	CLOA7	Canine	Labrador Retriever	14	FS	CLOA	FFPE	OD	2007	N/A	23,322
8	CLOA8	Canine	Bichon Frise	12	FS	CLOA	FFPE	OS	2007	N/A	28,670
9	CLOA9	Canine	Labrador Retriever	7	FS	CLOA	FFPE	OD	2007	N/A	30,838
10	CLOA10	Canine	Labrador Retriever	8.3	MN	CLOA	FFPE	OS	2008	N/A	25,659
11	CLOA11	Canine	Dalmatian	-	-	CLOA	FFPE	OD	2008	N/A	25,225
12	CLOA12	Canine	Australian Cattle Dog	6	FS	CLOA	FFPE	OD	2009	N/A	6096
13	CLOA13	Canine	Samoyed	11	FS	CLOA	FFPE	OS	2009	N/A	41,506
14	CLOA14	Canine	Shih Tzu	9	FS	CLOA	FFPE	OS	2009	N/A	26,233
15	CLOA15	Canine	Mix	16	MN	CLOA	FFPE	OD	2010	N/A	26,505
16	CLOA16	Canine	Shih Tzu	10	FS	CLOA	FFPE	OS	2010	N/A	4794
17	CLOA17	Canine	Cockapoo	8.5	FS	CLOA	FFPE	OS	2010	N/A	15,259
18	CLOA18	Canine	Labrador Retriever	12	FS	CLOA	FFPE	OS	2011	N/A	48,420
19	CLOA19	Canine	Labrador Retriever	-	MN	CLOA	FFPE	OS	2011	N/A	3561
20	CLOA20	Canine	Samoyed	10	FS	CLOA	FFPE	OS	2011		19,334
21	CLOA21	Canine	Toy Poodle	12	MN	CLOA	FFPE	OD	2012	Vaccinated for parvo and distemper, did not live w/ other pets	42,501
22	CLOA22	Canine	Boston Terrier	13	FS	CLOA	FFPE	OD	2012	N/A	27,442
23	CLOA23	Canine	Samoyed	13	FS	CLOA	FFPE	OD	2012	N/A	19,185

24	CLOA24	Canine	Labrador Retriever	11	FS	CLOA	FFPE	OS	2012	N/A	28,572
25	CLOA25	Canine	Labrador Retriever	8	FS	CLOA	FFPE	OS	2012	N/A	27,181
26	CLOA26	Canine	Shih Tzu	12	FS	CLOA	FFPE	OS, OD	2012	N/A	36,317
27	CLOA27	Canine	Miniature Poodle mix	9	MN	CLOA	FFPE	OS, OD	2013	N/A	33,507
28	CLOA28	Canine	Mix	10	FS	CLOA	FFPE	OD	2013	N/A	40,242
29	CLOA29	Canine	Golden Doodle	10	MN	CLOA	FFPE	OU (OD)	2016	N/A	64,801
30	CLOA30	Canine	Cockapoo	13	FS	CLOA	FFPE	OS	2016	N/A	33,687
31	CLOA31	Canine	Mix	11	FS	CLOA	FFPE	OS, OD	2007	N/A	28,972
32	PV1	Equine	-	8	MC	Sarcoid	FFPE	Medial canthus	2012	N/A	90,006
33	PV2	Canine	Labrador Retriever	10	FS	Papilloma	FF	Footpad	2017	N/A	72,626,435
34	PV3	Canine	Mastiff	F	2	Papilloma	FFPE	Skin	2011	N/A	8,209,598
35	PV4	Canine	Golden Retriever	M	5	Papilloma	FFPE	Lip	2012	N/A	28,014,256
36	NC1	Canine	Pitbull Terrier Mix	F	~3	Normal control	FF	Conjunctiva	2017	N/A	578
37	NC2	Canine	Shepherd/Pitbull mix	F	~4	Normal control	FF	Conjunctiva	2017	N/A	440
38	NC3	Canine	Pitbull mix	-	~5	Normal control	FF	Conjunctiva	2017	N/A	2996
39	NC4	Canine	Shepherd/Labrador retriever mix	-	~3	Normal Control	FF	Conjunctiva	2017	N/A	530
40	NC5	Canine	Mix	-	-	Normal Control	FF	Conjunctiva	2017	N/A	540

41	NC6	Canine	Lab/Shepherd/ Greyhound mix	F	9	Normal Control	FF	Conjunctiva	2017	History of Addison's disease	663
42	NC7	Canine	Jack Russell Terrier Mix	-	~2	Normal Control	FF	Conjunctiva	2017	N/A	269
43	NC8	Canine	Bouveir Des Flandres	FS	12	Normal Control	FF	Conjunctiva	2017	Metastatic hemangiosarcoma, Chemodectoma	866
44	NC9	Canine	Shepherd Mix	-	~7	Normal Control	FF	Conjunctiva	2017	N/A	725
45	NC10	Canine	Golden Retriever	MC	8	Normal Control	FF	Conjunctiva	2017	Duodenal plasmacytoma, megaesophagus, aspiration pneumonia	764

[a]Formalin fixed paraffin embedded (FFPE) or fresh frozen (FF)
[b]OS=left eye; OD= right eye
- Data unavailable
[c]Nt and nr reads

Table S2: Comparison of papillomavirus detection techniques with conventional PCR, IHC and ViroCap for positive control samples

Species	Sample	Lesion	FAP59/64 PCR	IHC[a]	ViroCap	Comments
Equine	PV1	Sarcoid	+	- Melanin in epithelium	+	BPV-1
Canine	PV2	Papilloma	+	+	+	CPapV-2. PCR typing done at Georgetown University.
Canine	PV3	Papilloma	-	+	+	CPapV-6
Canine	PV4	Papilloma	+	+	+	CPapV-1

[a]IHC: L1 stain

Table S3: FAP59/64 primer sequences and PCR conditions

Primer	Sequence	Amplicon	Conditions
FAP59 (forward)	TAACWGTNGGNCAYCCWTATT	480 bp	94°C 10 min,<br>45 cycles of:<br>94°C 1.5 min, 50°C 1.5 min, and 72°C 1.5 min<br>Final extension 72°C 5 min
FAP64 (reverse)	CCWATATCWVHCATNTCNCCATC		

Degenerate nucleotides: H = A, T, or C; N = A, G, C, or T; V = A, C or G; W = A or T; Y = C or T.
Final concentrations of the reaction products were ×1 PCR buffer, 1.5mM MgCl2, 200 μM dNTP, 0.25μM of each primer, 1.25 U platinum Taq DNA polymerase (Invitrogen Corporation, Carlsbad, CA, USA) and 50ng template DNA in a final reaction volume of 50μL.

Table S4: Parvovirus and Merkel Cell Polyomavirus primer sequences PCR conditions

Primer	Sequence	Amplicon	Conditions	PCR Reaction
**VP2**				
2655F*	CCAGATCATCCATCAACATCA	857 bp	98°C for 30 sec<br>35 cycles of:<br>98°C 10 sec, 62°C 30 sec, and 72°C 30 sec<br>Final extension 72°C 5 min	Final concentrations of the reaction products were 1x Phusion PCR buffer (cat #F530S; Thermo Scientific, USA), 0.2 mM dNTP, 0.5μM of each primer, 0.2 U Phusion High Fidelity DNA polymerase and 0.5ul template DNA in a final reaction volume of 20μL.
3511R*	TGAACATCATCTGGATCTGTACC			
3381F*	CCATGGAAACCAACCATACC	736 bp		
4116R*	AGTTAATTCCTGTTTTACCTCCAA			
**Large and Small T Antigen**				
LT3F	TTGTCTCGCCAGCATTGTAG	308 bp	95°C for 10 mins<br>40 cycles of:<br>95°C 15 sec, 56°C 1 min	Final concentrations of the reaction products<br>TB Green Advantage Premix (cat #639676; Takara Bio, USA)<br>10 ul, 10μM of each primer, 100ng template DNA in a final reaction volume of 20μL.
LT3R	ATATAGGGGCCTCGTCAACC			

*These primers allow differentiation between FPLV and CParvoV (overlapping fragments that amplify entire 1755 nucleotide VP2 region)

Table S5: Canine patients with parvovirus detected with ViroCap. Coverage metrics and closest parvovirus strain information are presented.

Sample	Read Count[a]	Closest match (GenBank Accession)[b]	DoC	BoC	Comments on coverage	Form	Comments
NC3	466	Canine parvovirus 2a strain CParvoV-YH, complete genome (KY403998.1)	12x	0.76%	Hairpin at the 3' end[c]	Linear	4923 bp genome. Deposited into GenBank as MZ647470.
CLOA21	272	Mink enteritis virus strain MEV-L, complete genome (KT899746.1)	7x	0.46%	Gaps in coverage in VP2, 3714-3784 bp and 4092-4157 bp.	Linear	Also close match to feline panleukopenia virus.
NC4	37	CParvoV-2b (EU659119.1)	1x	0%	Gaps throughout	N/A	N/A
CLOA2	47	Too few reads. Most consistent with MEV or FPLV	1.2x	0%	Gaps throughout	N/A	Not detected with PCR

[a]Reads that primarily mapped to the specified virus. Counts reported after deduplication with Picard.
[b]Closest match was determined by Sanger and Illumina sequencing
[c] The CParvoV-2a virus identified in NC3 contained reads in the 3' end of the genome that were in the inverted orientation. This is likely due to the rolling hairpin replication that occurs in parvovirus.

Table S6: Read counts for papillomavirus positive controls across all samples. One sample, PV4, had a read count that was above the 0.1% threshold and misalignment was suspected.

Virus	Total read count (ViroMatch)	Source of the CPapV	0.1% read threshold	Samples with CPapV reads above the threshold for contamination
Lambdapapillomavirus 2, CPapV-1	28,004,588	PV4	28,005	None
Taupapillomavirus, CPapV-2	73,043,821	PV2	73,044	None
Lambdapapillomavirus 3,	8,167,726	PV3	8168	PV4[a]

CPapV-6				
Deltapapillomavirus 4, BPV-1	60,281	PV1	60	None

[a] This was most likely a false positive since this sample was used in a previous study and CPapV-6 was not found in this sample [6]. Also, the genome of CPapV-6 identified in PV4 in this study was identical to the virus identified in PV3 and thus consistent with misalignment

Table S7: Coverage metrics and papillomavirus strains that were detected with ViroCap in the positive control samples

Sample	PV	GenBank Accession	Genera	Read count[a]	DoC	BoC	Physical form
PV2	Canine papillomavirus 2	MW881228	Tau	3,917,290	71,340x	100%	Circularized
PV4	Canine papillomavirus 1	KY825186.1	Lambda 2	2,300,847	37,928X	100%	Circularized
PV1	Bovine papillomavirus 1	KY886226.1	Delta	22,054	408x	82.25%	Circularized
PV3	Canine papillomavirus 6	KY802017	Lambda	1,209,109	20,761x	100%	Circularized

[a]Reads that primarily mapped to the specified virus. Counts reported after deduplication with Picard.

Table S8: Novel complete canine papillomavirus 2 genome discovered by ViroCap

Sample	New Genome Name	GenBank Accession	Genome Size	Closest relative (GenBank Accession)	Genera	% Analogous (genome)[a]	SBS[b]	Indels
PV2	Canine papillomavirus 2, isolate Missouri, Isabelle, complete genome	MW881228	8101	NC_006564.1	Tau	99.99%	1	0

[a]Percentage analogous was calculated with Clustal Omega

[b]Single base substitutions

[c]Reads that primarily mapped to the specified virus. Counts reported after deduplication with Picard.

Table S9: Variants and predicted functional outcome in the novel papillomavirus strain (GenBank MW881228) detected in PV2 compared to the reference canine papillomavirus 2 genome (NC_006564.1)

Strain	Nucleotide variant	Genomic Position	Gene	Amino Acid Variant	Variant Type	Domain/Feature	Provean variant score	Predicted Consequence
Canine papillomavirus 2, isolate Missouri, complete genome	A→G	7046	L1	K282R	Non-synonymous	N/A	-0.426	Neutral

Table S10: Canine patients with Merkel cell polyomavirus detected with ViroCap. Coverage metrics and closest Merkel cell polyomavirus strain information are presented.

Patients	Read Count[a]	Closest match (GenBank Accession)	DoC	BoC	Comments on coverage	Form
CLOA3	20	NC_010277.2	0.5x	0%	Gaps throughout	Circularized
CLOA31	50	HM011538.1	1.4x	3.69%	Gaps throughout	Circularized
CLOA1	35	NC_010277.2	0.9x	0%	Gaps throughout	Circularized
PV3	32	HM011538.1	0.9x	0%	Gaps throughout	Circularized
CLOA7	14	NC_010277.2	0.4x	0%	Gaps throughout	N/A
CLOA21	4	NC_010277.2	0.1x	0%	Gaps throughout	N/A
CLOA14	6	NC_010277.2	0.2x	0%	Gaps throughout	N/A
CLOA24	10	NC_010277.2	0.3x	0%	Gaps throughout	N/A
CLOA13	12	NC_010277.2	0.3x	0%	Gaps throughout	N/A
CLOA19	6	NC_010277.2	0.2x	0%	Gaps throughout	N/A
NC9	4	NC_010277.2	0.1x	0%	Gaps throughout	N/A
CLOA17	6	NC_010277.2	0.2x	0%	Gaps throughout	N/A
NC6	2	NC_010277.2	0.1x	0%	Gaps throughout	N/A
CLOA22	2	NC_010277.2	0.2x	0%	Gaps throughout	N/A

[a]Reads that primarily mapped to the specified virus. Counts reported after deduplication with Picard.

Figure S1: Aligned reads from the positive control sample PV2 that aligned to CPapV-2 (GenBank NC_006564.1) after deduplication are shown. This strain was deposited into GenBank as MW881228, Canine Papillomavirus 2, isolate Missouri, Isabelle, complete genome. Colored vertical lines on the grey coverage track represent single nucleotide variants compared with the reference.

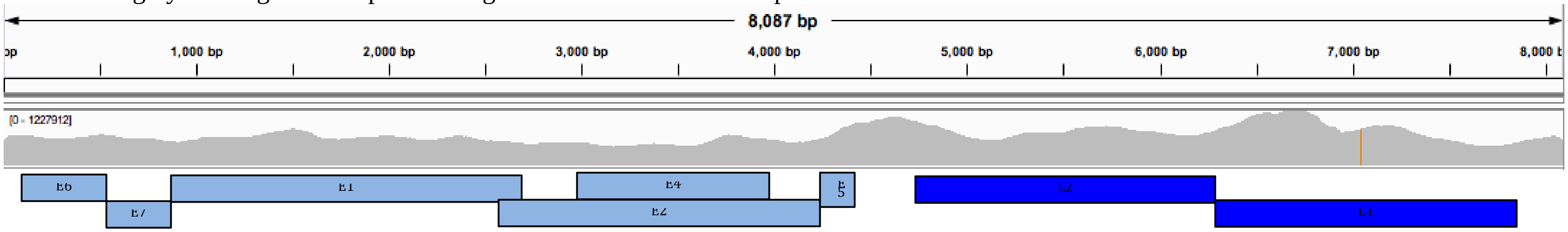

Figure S2: CLOA31 had the most reads that aligned to Merkel Cell Polyomavirus but the DoC and BoC were low. The 5343 bp Merkel Cell Polyomavirus (GenBank NC_010277.2) genome is annotated and shown above the reads that aligned to this genome. GP1 is the VP2 capsid protein. GP2 is the VP1 major capsid protein. GP3 is the large T antigen. The gene in the far right is the small T antigen.

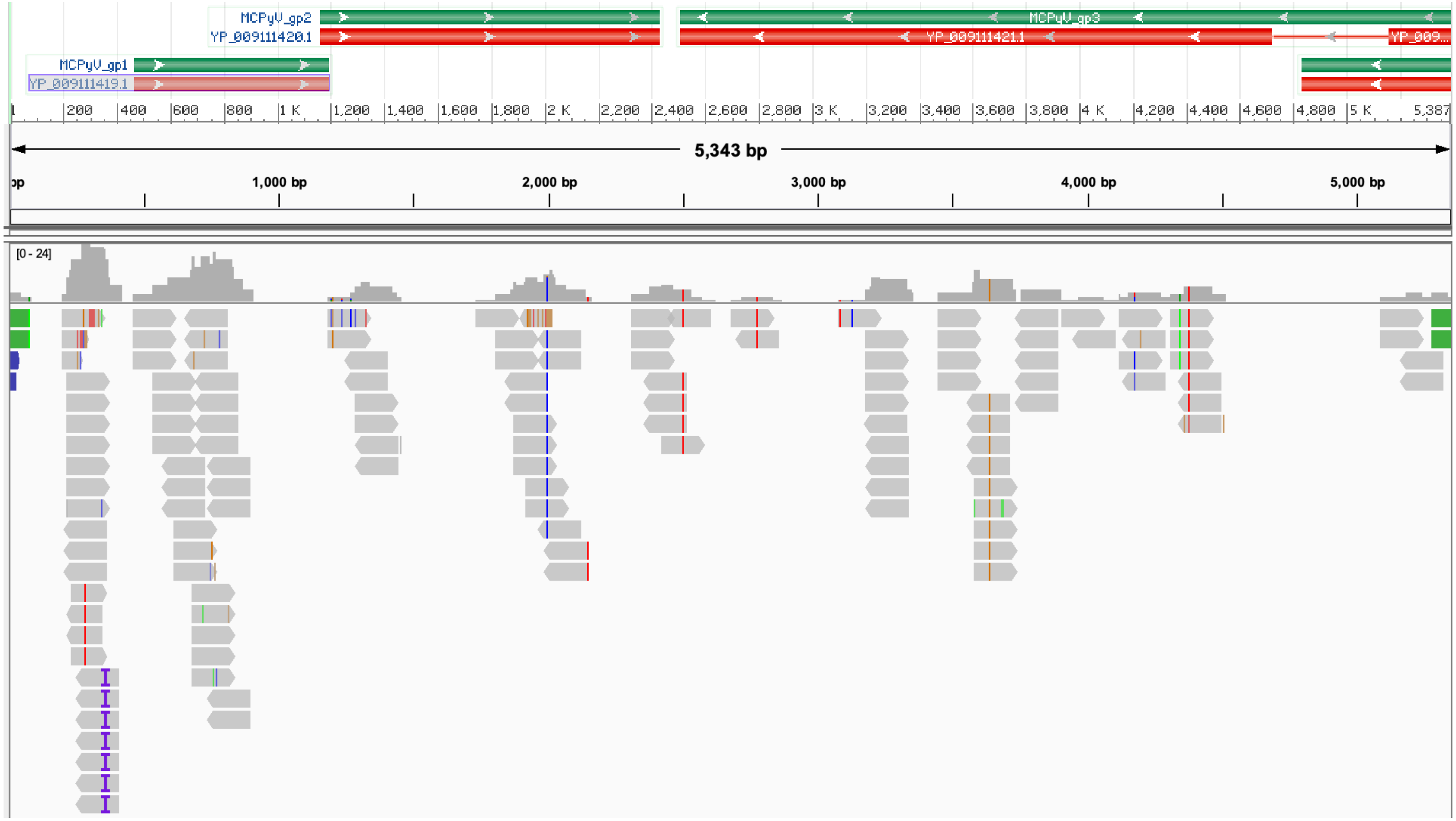

Figure S3: Merkel Cell Polyomavirus was detected by PCR and Sanger sequencing in the samples that contained the highest ViroCap Merkel Cell Polyomavirus read counts. The LT3 primer set was used. The PCR product was visualized on an 1% agarose gel as a 308 bp band. Lane 1: 100 bp ladder; lane 2: no template control; lanes 3-4: CLOA31 gDNA; lanes 5-6: CLOA31 library; lane 7: Merkel Cell Polyomavirus plasmid pcDNA6.TAg206.V5(2B4); lane 8: Merkel Cell Polyomavirus plasmid pcDNA3.MCV339 (144-3696); lanes 9-10: CLOA1 gDNA; lanes 11-12: CLOA3 gDNA that was submitted for library production and sequencing; lanes 13-14: CLOA3 gDNA that was not submitted for library production and sequencing; lanes 15-16: PV3 gDNA. These amplicons were subsequently sequenced via Sanger sequencing and confirmed the presence of Merkel Cell Polyomavirus in lanes 7-8 (Merkel Cell Polyomavirus positive control plasmids), lane 5 (CLOA31 library), lane 12 (CLOA3 gDNA submitted sample) and lanes 15-16 (PV3 gDNA).

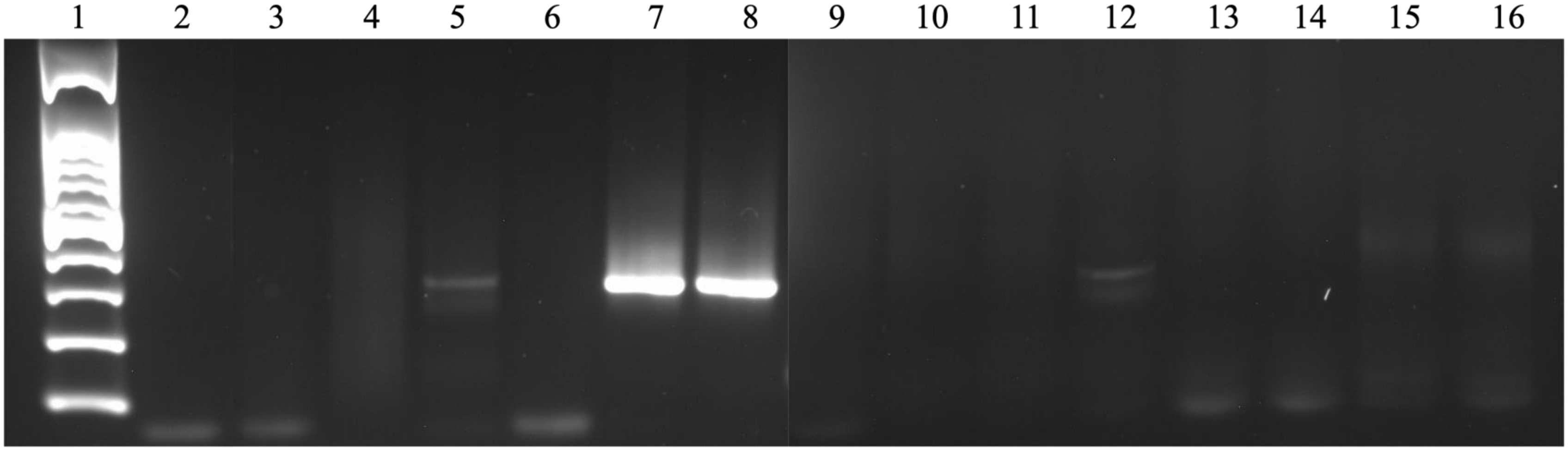

References

1. Costello, M.; Fleharty, M.; Abreu, J.; Farjoun, Y.; Ferriera, S.; Holmes, L.; Granger, B.; Green, L.; Howd, T.; Mason, T.; et al. Characterization and remediation of sample index swaps by non-redundant dual indexing on massively parallel sequencing platforms. *BMC Genomics* **2018**, *19*, 332, doi:10.1186/s12864-018-4703-0.
2. Wylie, K.M.; Wylie, T.N.; Buller, R.; Herter, B.; Cannella, M.T.; Storch, G.A. Detection of Viruses in Clinical Samples by Use of Metagenomic Sequencing and Targeted Sequence Capture. *J Clin Microbiol* **2018**, *56*, doi:10.1128/JCM.01123-18.
3. Kircher, M.; Sawyer, S.; Meyer, M. Double indexing overcomes inaccuracies in multiplex sequencing on the Illumina platform. *Nucleic Acids Res* **2012**, *40*, e3, doi:10.1093/nar/gkr771.
4. Kircher, M.; Heyn, P.; Kelso, J. Addressing challenges in the production and analysis of illumina sequencing data. *BMC Genomics* **2011**, *12*, 382, doi:10.1186/1471-2164-12-382.
5. Sinha R, S.G., Gulati GS, Ezran C, Travaglini KJ, Wei E, Chan CKF, Nabhan AN, Su T, Morganti RM, Conley SD, Chaib H, Red-Horse K, Longaker MT, Snyder MP, Krasnow MA, Weissman IL. Index switching causes "spreading-of-signal" among multiplexed samples in Illumina HiSeq 4000 DNA sequencing. *bioRxiv* **2017**, doi:http://dx.doi.org/10.1101/125724.
6. Chu, S.; Wylie, T.N.; Wylie, K.M.; Johnson, G.C.; Skidmore, Z.L.; Fleer, M.; Griffith, O.L.; Bryan, J.N. A virome sequencing approach to feline oral squamous cell carcinoma to evaluate viral causative factors. *Vet Microbiol* **2020**, *240*, 108491, doi:10.1016/j.vetmic.2019.108491.
